# Regional coronary endothelial dysfunction is related to local epicardial fat in HIV+ patients

**DOI:** 10.1186/1532-429X-18-S1-O13

**Published:** 2016-01-27

**Authors:** Allison Hays, Micaela Iantorno, Michael Schär, Sahar Soleimanifard, Richard Moore, Gary Gerstenblith, Robert Weiss

**Affiliations:** 1 Medicine, Johns Hopkins, Clarksville, MD USA; 2 grid.21107.350000000121719311Radiology, Johns Hopkins, Baltimore, MD USA

## Background

With improved survival resulting from anti-retroviral therapies, accelerated coronary artery disease (CAD) has become an important cause of significant disability and premature death in HIV-infected patients. HIV+ people often accumulate epicardial adipose tissue (EAT), a metabolically-active visceral fat capable of releasing inflammatory adipokines that could contribute to CAD through local paracrine actions. We tested the hypothesis that abnormal coronary endothelial function (CEF), an early marker of atherosclerosis and predictor of CAD events, is related to the amount of local pericoronary EAT in HIV+ patients.

## Methods

We studied 30 HIV+ subjects, 16 with no CAD ("HIVnoCAD", 0 Calcium score by CT, mean age: 53 ± 3 years, 22 RCA segments) and 14 with known CAD ("HIV CAD", 58 ± 4 years, 19 segments). To measure CEF, coronary MRI was performed before and during isometric handgrip exercise (IHE), an endothelial-dependent stressor and % coronary cross-sectional area (CSA) change was quantified. MRI for coronary EAT local area and thickness in the atrioventricular groove was performed at the same imaging plane as that used for CEF. All data are expressed: as mean ± SEM.

## Results

In HIVnoCAD subjects**,** the IHE-induced % change CSA was significantly greater than in HIV CAD subjects (3.7 ± 1.2% v. -1.0 ± 1.0%, p = 0.004) and mean pericoronary EAT area was lower, 209 ± 16 mm^2^ v. 293 ± 31 mm^2^, p = 0.02). There was a significant inverse relationship between % CSA change with IHE and both local fat area (R = -0.42, p = 0.007) and fat thickness (R = -0.60, p < 0.0001) for all HIV subjects.

## Conclusions

The extent of local coronary EAT is significantly related to coronary endothelial dysfunction and thus may be an important contributor to coronary disease in HIV+ patients. Interventions to reduce this component of visceral adipose tissue may favorably limit the otherwise accelerated coronary atherosclerosis in this important patient population.

**Figure 1:**
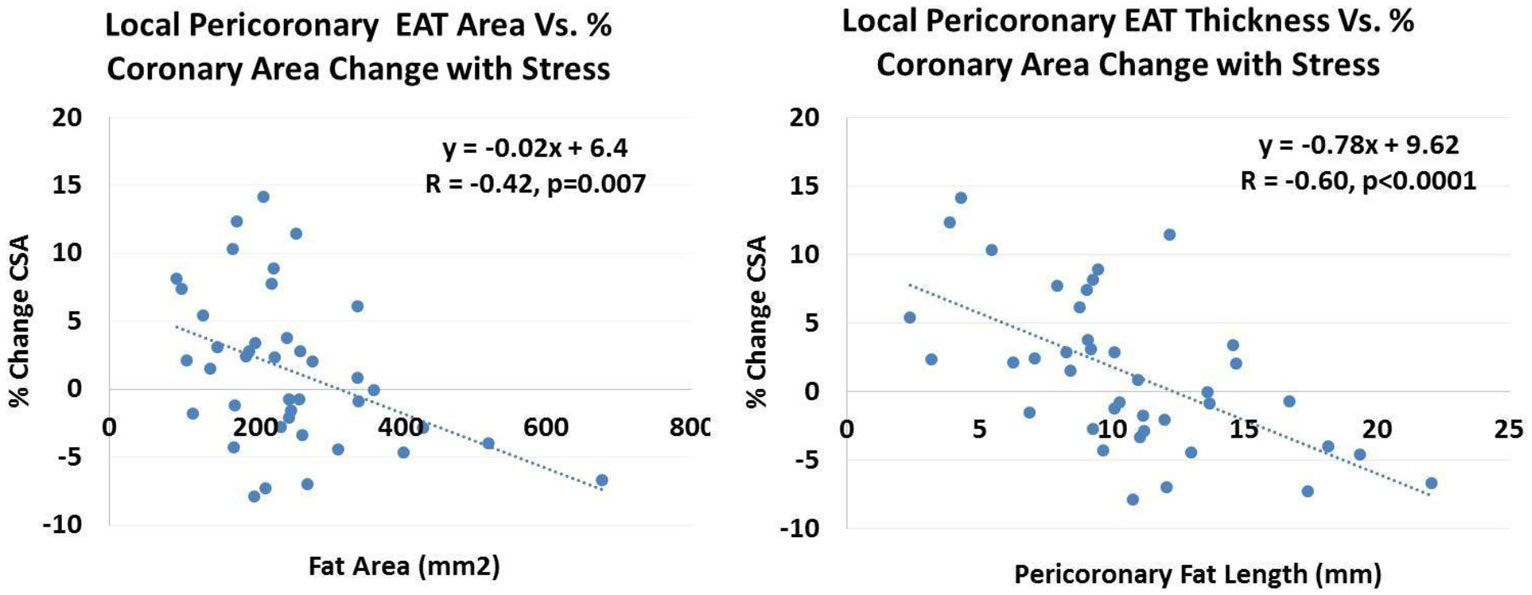
**Degree of local (pericoronary) EAT is inversely related to coronary macrovascular endothelial function (% CSA (coronary cross sectional area) change with isometric handgrip stress) in HIV patients (N = 41 coronary segments in 30 HIV patients).**

